# The Truth Shall Make Us Free

**Published:** 1895-03

**Authors:** J. H. Allen

**Affiliations:** Logansport, Ind.


### "THE TRUTH SHALL MAKE US FREE."

J. H. Allen, M. D., Logansport, Ind.

"He is a freeman whom truth makes free, and all are slaves beside." "Seek the truth: come whence it may, cost what it will."

The title of my paper may be said to be universal in its application. The truth alone can make us free. "True it is that knowledge comes, but wisdom lingers." Knowledge may be anything, but truth is wisdom. We theorize and we philosophize, and we draw geometrical lines and angles with wonderful acuteness, but frequently come wide of the mark. We are creatures of limitation, very much so, as we are only brought into relationship with a small part of this universe, therefore our reasoning, and our theorizing, and our philosophizing are in vain if we do not do so from some fixed principle. All knowledge not based upon some fixed principle, some law, is sure to be an uncertainty, and being an uncertainty it breeds empiricism, and empiricism, though it may have plausibility on the face of it to the casual observer, to the scientific mind, which analyzes its phenomena by applying some fixed law or principle, it loses that plausibility and becomes a thing impossible. And when we let in the search-light of truth upon empiricism we find chaos in place of cosmos, disorder and dissolution follow in its footsteps, and, like all chaos, it is constantly seeking a centre around which to revolve. Its relationship to a science is inharmonious, unsymmetrical, disconnected.

Truth's forerunner is light; its sky is sunshine; there are no clouds nor misty exhalations in its atmosphere, but a clearness and distinctness that can but be appreciated by one who has the light. It enables the true follower of Hahnemann to render unto Cæsar that which is Cæsar's, and unto Homœopathy that which belongs to it. If the principle is based upon truth its developments are light, cosmos, and harmony; but, on the other hand, if the so-called science is based upon man's empiricism, then surely chaos and confusion would follow, dissolution and death be its offsprings.

"More light, more light!" cried Goethe, the great poet, at his death, and the world has ever since been voicing the cry, but it has ended with nothing but a cry. Then let us get hold of the first principle of the universe, *truth*, and it will kindle in our minds a sun that will brighten up the dark places and give us a conception of things that are divine, and so broaden our mental field of vision that we may analyze them, not only through the gross materialism of our senses, but through the great avenues of the mind.

Our knowledge of law puts the keys of the universe in our possession and opens the shining doors of a true science. Being freed from darkness by the light of truth, our feet are placed upon the platform of the positive, and they who have the light, walk as a man having no fear, his step is one of firmness, his demeanor calm, and his presence instills into the mind of the sick and suffering one confidence, peace, and rest. He is a man of law, therefore, a man of power. Empiricism, which once held him as a slave, now, through the new light and his knowledge of law, he is made free; his words are yea, yea, and nay, nay. He looks upon disease not through some name of man's prefix, around which are situated etiologies, times, sequels, stages, and conditions, but he looks upon the phenomena of diseases and meets those phenomena by other phenomena, which he draws upon from the different well-proven drugs. In the potent drop he sees his patient loosed from the bonds of pain, suffering, and death. Thus he reasons, if it is the truth it needs no expedient. A local application of heat or cold will not intensify its action. A poultice, a blister, or counter-irritant will not assist it in its dynamic contest with the forces that are battling for supremacy within. The cure cannot come from the mechanical forces from without, but by the dynamic forces within, aided by the magic touch of similia. While, on the other hand, we must naturally drift into empiricism, having no clear conception of law so as to apply similia. You see expedience would be a natural consequence, make-shifts follow such uncertainty, and substitution be the natural order of things. "Here the truth half told becomes ever the blackest of lies," and the physician becomes a knave and a juggler in place of a man of science. I would not insult my readers by fully comparing him with this man of science save in a general way. In the first place we have order, in the second chaos; certainty in the one and uncertainty in the other. One is positive, the other is negative. One is acquainted with the truth and can apply it; the other knows not the truth, and, therefore, substitutes something for the truth.

Again, truth frees us from the bondage of custom; it was, and still is, to some degree, the custom to bleed, to blister, to cauterize, and to vaccinate, and the day was when custom would seem to demand it of us. But our knowledge of the truth of pure Homœopathy came forward in the power of its omnipotence and said, *no!* When we follow custom we wear the grave-clothes of the dead, we are skeletons in their old armor—in fact, we become hero-worshipers of isms and issues that should have been buried in the sarcophagus of their originators. Then let us follow no longer men who, though honest in their endeavors, but lacking any knowledge of law, have conjured up these expedients and make-shifts in the vain hope of mitigating the cry of woe that has followed the scourge of death.

Again, truth frees us from the fear of man and his criticisms. What is the truth? They know not, and, *because* they know not, they drift into rationalism and bitterly oppose the truth. But what cares he who knows the truth? no more than did the martyrs of old care for the frenzied mob or maddening crowd, nor for fire or torturing rack, nor for life or death in whom the truth made free.

Again, the truth frees us from the unscientific methods of our day—from the bandages, the wrappings, the splints, the nomenclatures of a school of medicine which, in place of being governed by law, is governed by license. It unincumbers us from these things, so that we throw off the clods of materialism and rise into the higher altitudes of the true knowledge of Homœopathy, and are possessors off the privileges of men of science who are governed by the supremacy of law. Some one asked Spurgeon, after a splendid address, why he did not say something about science in his discourse, and his answer was, "I have not read the morning paper." So we might say of the dominant school of medicine to-day, we do not know what they believe in, because we have not read their latest journal. I have in my mind compared their history to the flight of the firefly—a faint spark of light, then a long pause of utter darkness, then again a momentary flash, a hopeful gleam of light amid the dark, but in a moment we have night again, and greater and denser is that night because our eyes were dazzled by the glare.

In the old adage, that "Truth is mighty and will prevail," we have some hope and much promise in the stalwart march of the young giant during the past hundred years.

Once more truth places us under the guidance of a physical law of the universe, and we at once become laborers in truth in restoring harmony, because law brings harmony and restores order in every system over which it has control, then our knowledge of truth places us in the proper position to watch and study the development of law, or, rather, its phenomena, and when new developments make their appearance, we place them in their order and in their proper relationship to that law, with due reverence and not with a feeling of a Columbus, as in the case of Pasteur, Koch, and Behring, thinking they have discovered a new law, when, simply by accident, they have developed a part of the phenomena of a great principle, known only to the followers of Hahnemann, who alone are able to apply those phenomena as it may develop in its true relationship to that law, and so on *ad infinitum.*

Finally, truth not only reveals the present condition of the sick and suffering one in the true light, but, by having a true knowledge of the law of homœopathics, we can look down through that winding channel in which the sick one has passed and by a careful analysis of his symptoms can trace the colored strand of disease that has been woven into the web of life, first, probably, in psora or latent syphilis in the state primeval, and the patient have all the appearance of health and long life, kept under control or in a quiescent state by the superior gravitational forces of the young life, until the disturbing element came, in some pacific form, and the attending physician, not knowing the nature of this disturbing element, save in name, began a course of action against it that was not in harmony with natural law, and in place of eliminating through natural law, he, through his ignorance of the truth, suppressed it, and what is the result? Dare I tell to the world that that physician, in place of being a guardian angel of life, did at that moment plant the seeds of death, and, if he could have seen in the light of the living truth, that a destructive process began at that moment which robbed him of his greatest earthly blessing, health, and either hurried him on the road to death or made life a living sacrifice to disease.

